# Possible contribution of quantum-like correlations to the placebo effect: consequences on blind trials

**DOI:** 10.1186/s12976-017-0058-5

**Published:** 2017-06-02

**Authors:** Francis Beauvais

**Affiliations:** 91, Grande Rue, Sèvres, France

**Keywords:** Placebo effect, Quantum-like correlations, Experimenter effect, Randomized clinical trials

## Abstract

**Background:**

Factors that participate in the biological changes associated with a placebo are not completely understood. Natural evolution, mean regression, concomitant procedures and other non specific effects are well-known factors that contribute to the “placebo effect”. In this article, we suggest that quantum-like correlations predicted by a probabilistic modeling could also play a role.

**Results:**

An elementary experiment in biology or medicine comparing the biological changes associated with two placebos is modeled. The originality of this modeling is that experimenters, biological system and their interactions are described together from the standpoint of a participant who is uninvolved in the measurement process. Moreover, the small random probability fluctuations of a “real” experiment are also taken into account. If both placebos are inert (with only different labels), common sense suggests that the biological changes associated with the two placebos should be comparable. However, the consequence of this modeling is the possibility for two placebos to be associated with different outcomes due to the emergence of quantum-like correlations.

**Conclusion:**

The association of two placebos with different outcomes is counterintuitive and this modeling could give a framework for some unexplained observations where mere placebos are compared (in some alternative medicines for example). This hypothesis can be tested in blind trials by comparing local vs. remote assessment of correlations.

## Background

Much has been written about the “placebo effect” and the purpose of this article is not to make a review on this topic [1–6]. In itself the term “placebo effect” is curious and paradoxical. Indeed, as underscored by Moerman and Jonas: “*The one thing of which we can be absolutely certain is that placebos* do not *cause placebo effects. Placebos are inert and don’t cause anything”* [1]. For this reason, Ernst and Resch insisted to clarify the definition of placebo by distinguishing “perceived placebo effect” and “true placebo effect” [7]. Perceived placebo effect is the outcome that is associated with the placebo group in a trial; it includes natural evolution of the disease, mean regression, concomitant procedures and other non specific effects. True placebo effect is the difference between perceived placebo effect and effect associated with no treatment. “No treatment” groups are however infrequently performed and therefore there are often some misunderstandings to define the scope of the “placebo effect”.

Since placebos are inert, the causes of the “true placebo effect” should be sought rather on the side of language and psychology. Thus, it has been shown that placebo effects can be caused by cognitive and emotional changes, expectation of symptom changes or classical conditioning [2]. Actual effects of placebo on brain and body have been evidenced and there are neurobiological underpinnings for these effects [8, 9].

However, in most studies aimed to decipher the placebo effect, patients are at the centre of the investigations and all explanations rest on them. In the present article, the experimental design and the experimenters are also taken into consideration. Therefore, the focus is moved from patients to investigators and in this case the placebo effect – at least one of its components – is not much different than an experimenter’s effect. A famous example of experimenter’s effect was evidenced in the experiments of Rosenthal et al. where an experimenter obtained from his subjects the data he expected or wanted to obtain [10]. Outside of psychology, for example in cell biology or in physiology, it is generally thought that such subtle influences could not be responsible for response biases. In clinical trials, blind experiments are supposed to protect against any outcome bias related to patient or physician; if such influences exist, they are distributed randomly in test and placebo groups. In the present article, it is suggested that quantum-like correlations predicted by a probabilistic modeling could also contribute to the “placebo effect”.

## Results

### Design of a minimal experiment with two placebos

The purpose of a typical experiment in medicine or biology is to establish a relationship between a “cause” (independent variable) and a biological “effect”. Placebos (or “controls” in experimental biology) are included in the experiment in order to assess the effects of variables other than the independent variable.

We define a biological “object” (biological model or patients in a clinical trial) with two possible states: no biological change (or resting state, not different from background noise) and biological change (“activated” state). A biological change may be defined by setting a cut-off value of a continuous variable. We symbolize no biological change as “↓” and biological change as “↑”.

We assume that all samples that are tested are placebos and that the only difference is their labels which are either *Pcb*_0_ or *Pcb*_1_. Since samples are all inert and physically identical, common sense suggests that the biological outcomes associated with the two placebos should be comparable. Nevertheless, the aim of the modeling is to know whether in some circumstances the state “↑” could be more frequently observed with one of the two labels (no matter which one at this stage). Therefore, the null hypothesis (H_0_) of such an experiment is:

1 $$ {\mathrm{H}}_0:\mathrm{Prob}\ \left(\uparrow \Big| Pc{b}_0\right)=\mathrm{Prob}\left(\uparrow \Big| Pc{b}_1\right) $$

Prob (*x*∣*y*) is the conditional probability of *x* given *y* (or the probability of *x* under the condition *y*).

**Fig. 1:**
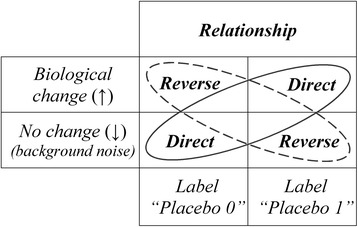
Relationships between placebos and biological system. There are two possible placebos (“0” and “1”) and two possible states for the biological system: no change (“resting” state or background) which is noted “↓” and biological change above background (“activated” state) which is noted “↑”. As a consequence, there are two possible relationships defined as: direct relationship with “placebo 0” associated with “↓” and “placebo” 1 associated with “↑”; reverse relationship with “placebo 0” associated with “↑” and “placebo 1” associated with “↓”

Figure 1 describes the two possible relationships between labels and biological outcomes: either “direct” relationship (*Pcb*_0_ associated with “↓” and *Pcb*_1_ associated with “↑”) or “reverse” relationship (*Pcb*_0_ associated with “↑” and *Pcb*_1_ associated with “↓”). Note that this naming (“direct” or “reverse”) is arbitrary and does not prejudge results. Of course, if a biological change is associated with the labels *Pcb*_0_ and *Pcb*_1_ with the same rate (i.e. no relationship), then the probabilities of direct and reverse relationship are both equal to 1/2. By convention, we present calculations of probability mainly for direct relationship (the sum of the probabilities of direct and reverse relationships is equal to one).

### Description of an experiment from an uninvolved standpoint

The originality of the present modeling is that observers, observed system and their interactions are described together from an uninvolved standpoint. The formalism is inspired from the relational interpretation of quantum physics [11, 12] and quantum Bayesianism (QBism) [13, 14].

**Fig. 2:**
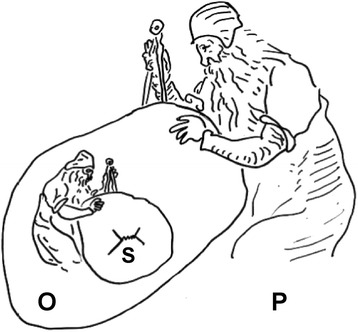
Description of an experiment from an uninvolved standpoint. The observer *O* measures the system *S* whereas the participant *P* remains uninvolved in the measurement (he does not interact with *O* and *S*). *P* knows that *O* has observed a definite state of *S*, but he does not know which one. If *P* finally interacts with *O* and *S*, then *P* and *O* agree on the outcome of *S*. The reasoning can be continued with another participant *Q* who does not interact with *S*, *O* and *P*. What *Q* can say is that *S*, *O* and *P* have definite values that are correlated. The only thing that an uninvolved participant can do is to describe the form, but not the content of the information available to the observers who interact with *S* and with each other. Thus, the consistency of any measurement is guaranteed (*GNU Free Documentation License*)

We suppose that the experimental landscape is described by a participant who is uninvolved in the experiment. Suppose, as described in Fig. 2, an observer *O* who measures a variable of a system *S*; this variable can take one of the two values “left” and “right” after a measurement. For a participant *P* uninvolved in the measurement process, a definite value has been obtained after the measurement of *S* by *O* (either “left” or “right”). *P* knows *that O* has observed a defined value after measurement, but *P* does not know *what O* has observed. If *P* finally observes the system *S*, he records a definite value and he agrees with *O* on this value when *P* and *O* interact. Interactions between observers are like measurements and they allow establishing correlations.

In this last case, it is important to underscore that it is not correct to say that *P* is “forced” to observe what *O* observed before they interact. Indeed, one can imagine another participant *Q* who in turn describes *S*, *O* and *P* without interacting with them. What *Q* can say is that a correlation has been established between *S*, *O* and *P*, but *Q* cannot say which result is observed. The only thing that an uninvolved participant can do is to describe the *form* (correlations), but not the *content* (outcomes) of the information available to the observers who interact with *S* and with each other. Thus, the consistency of any measurement is guaranteed.

Note that, strictly speaking, an uninvolved participant does not describe the “reality” itself (made of “contents”), but he constructs a predictive tool (made of correlations and probabilities) in order to know what to expect if he decides to interact with the “real” observers.

### Probabilistic modeling of a minimal experiment with two placebos

An experiment is modeled from the standpoint of a participant *P* who is outside the laboratory, as described in the previous section. The participant *P* does not interact with the “objects” that he describes and he remains uninvolved in the evolution of the experimental situation. The role of *P* is to describe the evolution of a *team of interacting experimenters* with the knowledge of the initial conditions.

We consider a team composed of two experimenters named *O* and *O’* who observe the biological system *S*. We suppose an experimental situation where the probability for each experimenter to observe a direct relationship (as defined in Fig. 1) is *p* and the probability of a reverse relationship is *q* (with *p* + *q* = 1).

Each observer has his own probabilistic expectations and the uninvolved participant *P* assigns the probability *p* to *O* as the best estimate that *O* can make for the future observation of the direct relationship. The same probability *p* is assigned to *O’* independently of *O* since the probabilistic expectations are specific to each observer.

**Fig. 3:**
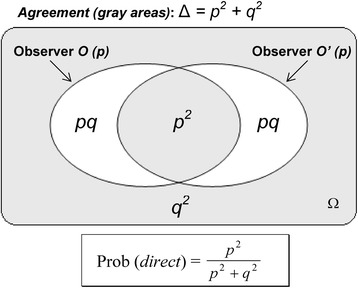
Probabilistic space constructed by an uninvolved participant *P* to predict the outcomes of the experiments. A team of interacting experimenters *O* and *O’* is described from the standpoint of an uninvolved participant who knows the initial experimental conditions (Fig. 2). We suppose a probability equal to *p* for the event “direct relationship” and equal to *q* for the event “reverse relationship” (*p* + *q* = 1). Each observer has his own probabilistic expectations and *P* assigns the probability *p* to *O* as the best estimate that *O* can make for the future observation of a direct relationship; the same probability is assigned to *O’ independently* of *O* since the probabilistic expectations are specific to each observer. White areas are unauthorized experimental situations with incompatible outcomes after interaction of *O* and *O’* (e.g. “direct” for *O* and “reverse” for *O’*). Therefore, the probability that the experimenters observe a direct relationship is calculated by dividing the central gray area (“direct” for both observers) by the sum of the probabilities of possible outcomes (either “direct” or “reverse” for both observers), namely all gray areas. Ω, probability space

Therefore, the participant *P* constructs a predictive tool for his own use where the experimenters have independent probabilistic expectations on the experimental outcome and are in agreement when they compare their records. The future outcome that *O* expects to record (event *A*) and the future outcome that *O’* expects to record (event *B*) are *independent* events in the probability space constructed by *P* (Fig. 3). This condition of independence is easily formalized since the probabilities of two independent events *A* and *B* have well-known mathematical properties:

2 $$ \mathrm{Prob}\left( A\cap B\right)=\mathrm{Prob}(A)\times \mathrm{Prob}(B) $$

Therefore, when *O* and *O’* interact and agree on the result of the experiment (i.e. the events of the set *A* ∩ *B*), the best estimate of the probability that *O* and *O’* observe a direct relationship is Prob (*direct*) = *p* × *p* according to Eq. (2) since the probability to record a direct relationship was estimated to be *p* for *O* and also *p* for *O’* (Fig. 3). Similarly, the best estimate of the probability that *O* and *O’* observe a reverse relationship is Prob (*reverse*) = *q* × *q*.

The intersubjective agreement discards some impossible situations such as *O* observes a direct relationship while *O’* observes a reverse relationship. Since the sum of the probabilities of all possible events is equal to one, Prob (*direct*) = *p* × *p* must be renormalized. For this purpose, *p* × *p* is divided by the sum of the probabilities of all possible outcomes (grey areas in Fig. 3), namely direct relationship (*p* × *p*) and reverse relationship (*q* × *q*):

3 $$ \mathrm{Prob}\ (direct)=\frac{p^2}{p^2+{q}^2} $$

By dividing both the numerator and the denominator by *p* ^*2*^ (taking into account that *p* + *q* = 1), the only variable of the equation is *p*:

4 $$ \mathrm{Prob}\ (direct)=\frac{1}{1+{\left(\frac{1}{p}-1\right)}^2} $$

Eqs. (3), (4) and Fig. 3 are easily generalized to any number *N* of observers who all agree on the outcome:

5 $$ \mathrm{Prob}\ (direct)=\frac{1}{1+{\left(\frac{1}{p}-1\right)}^N} $$

In a “real” experiment, particularly in biology, random fluctuations occur and they must be taken into account because, after each elementary fluctuation, a tiny bias is introduced and Prob (*direct*) must be updated.

In the next lines we calculate the evolution of the probability for *O* and *O’* to observe a direct relationship from the standpoint of the participant *P.* First, we write that Prob (*direct*) is equal to 1/2 in the absence of observers (*N* = 0 in Eq. (5)). As a consequence, the initial value of Prob (*direct*) at time *t*_0_ before the first fluctuation is equal to *p*_0_ = 1/2.

We then introduce ε_i_ as successive elementary random fluctuations of Prob (*direct*) that occur during successive elementary intervals of time (ε_i_ are positive or negative real random numbers such as ∣ε_i_∣ < < 1). Note that an implicit consequence of the random fluctuations of Prob (*direct*) is a non-null, but very small, probability to observe a biological change (“↑”).

After the first fluctuation ε_1_, we easily calculate with Eq. (4) the updated probability *p*_*1*_ which is based on *p*_0_ + ε_1_. The equation is then generalized for any probability *p*_*n+1*_ based on previous probability *p*_*n*_ and fluctuation ε_n+1_. We obtain a mathematical sequence which allows calculating the successive probabilities of a direct relationship:

6 $$ {\mathrm{Prob}}_{\mathrm{n}+1}\kern0.35em ( d i r e c t)={p}_{\mathrm{n}+1}=\frac{1}{1+{\left({\frac{1}{p_{\mathrm{n}}+\varepsilon}}_{\mathrm{n}+1}-1\right)}^N}\kern0.35em \mathrm{with}\kern0.35em {p}_0=1/2 $$

### Two placebos associated with different outcomes

Equation 6 allows calculating the successive states of a system constituted of a biological system and a team of interacting experimenters/observers committed in the establishment of a supposed relationship.

**Fig. 4:**
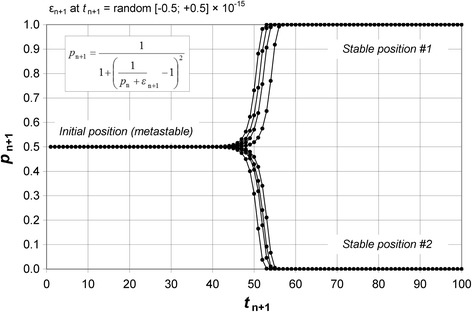
Calculation of the probability of a direct relationship. The evolution of the probability that a team (composed of two members who interact) observes a direct relationship is described in this figure by taking into account successive probability fluctuations. The probability defined in Fig. 3 is calculated according to the mathematical sequence in the cartouche. Each successive probability *p*_n+1_ of the sequence is calculated by using *p*_n_ and a random probability fluctuation is randomly obtained between −0.5 and +0.5 × 10^-15^. This computer simulation shows that the initial state with a probability of 1/2 is in fact metastable and, after a dramatic transition, one of two stable positions is achieved: either Prob (*direct*) = 1 or Prob (*direct*) = 0. With *N* > 2 or with higher values of probability fluctuations, a transition is obtained after a lower number of calculation steps (data not shown). Eight computer simulations are reported in this figure

A computer calculation of this mathematical sequence is described in Fig. 4 after 100 successive random fluctuations ε_i_ (with values around 10^−15^) and with two observers (*N* = 2). We observe that the initial situation is in fact *metastable* if fluctuations are taken into account. Indeed, in all cases (i.e. whatever the series of values ε_i_), a dramatic transition towards one of two stable positions is achieved:

7 $$ \begin{array}{c}\kern1em \mathrm{Prob}\kern.35em \left(\mathrm{direct}\right)=1/2\kern.35em \left(\mathrm{metastable}\kern.35em \mathrm{position}\right)\kern1em \\ {}\kern1em \downarrow \kern1em \\ {}\kern1em \mathrm{Prob}\kern.35em \left(\mathrm{direct}\right)=1\kern.5em  or\kern.5em 0\kern.35em \left(\mathrm{two}\ \mathrm{possible}\kern.35em \mathrm{s}\mathrm{table}\kern.35em \mathrm{position}\mathrm{s}\right)\kern1em \end{array} $$

All samples of an experiment are thus engaged either in a direct relationship or in a reverse relationship. Note that the probability of a biological change was very small initially and, after the transition, a biological change is systematically associated with the label *Pcb*_1_ in stable position #1 or systematically associated with the label *Pcb*_0_ in stable position #2. The choice of one of the two stable positions is random. In both cases, a relationship (direct or reverse) between labels and outcomes emerges.

However, the purpose of an experiment is to compare a “test” situation with a “control” situation. Biological systems are therefore prepared in an asymmetrical state with resting state (background noise) implicitly associated with a “control” situation. The stability of the resting state (or basic state) is a condition for a proper assessment of the samples (the experiment begins with the preparation of the biological system before samples are tested). In other terms, the state of the biological model at rest (before each test) can be considered as associated with the label “control”.

We suppose, for example, that *Pcb*_0_ is considered as a “control” by the experimenters. Consequently the stable state #2 eliminates itself since *Pcb*_0_ cannot be associated both with change (when *Pcb*_0_ samples are tested) and with no change (for the resting state). Only the stable position #1 is a possible state:

8 $$ \begin{array}{c}\kern1em \mathrm{Prob}\kern.35em (direct)=1/2\kern.35em \left(\mathrm{metastable}\kern.35em \mathrm{position}\right)\kern1em \\ {}\kern1em \downarrow \kern1em \\ {}\kern1em \mathrm{Prob}\kern.35em (direct)=1\kern.35em \left(\mathrm{stable}\kern.35em \mathrm{position}\right)\kern1em \end{array} $$

A probability equal to one for the direct relationship means that the participant *P* is assured – if he finally interacts with the team of experimenters after the end of the experiment – to observe a direct relationship between labels and biological outcomes. Thanks to probability fluctuations, a biological change associated with each sample with *Pcb*_1_ label emerges from background noise.

### Consequences of different types of blind designs on correlations

Until now we examined an experimental situation where the observers *O* and *O’* assessed themselves the rates of correlation between labels and biological outcomes (open-label experiments). Nevertheless, the labels can be masked to the experimenters in order to reduce or eliminate any bias. After the outcomes have been obtained in blind conditions for all samples, the labels of samples are unmasked. In this article, we distinguish blind experiments with either local or remote assessment of correlations.

For a local assessment of correlations with blind design, an automatic device or a member of the team of experimenters keeps secret the labels of the samples until the end of the experiment. In this case, the automatic device or the observer who is dedicated to the blinding are also elements of the experiment because they interact with the other observers and can be described (from the standpoint of *P*) with the same modeling as open-label experiments.

A remote assessment of correlations with blind design is typically used in randomized clinical trials (also named centralized blind design). The remote supervisor (a statistician for example) does not interact with the experimenters before all measurements are completed. It is important to underscore that the remote supervisor should not be confused with the uninvolved participant *P* who describes the experiment. Indeed, *P* does not interact and is not involved in the experiment. With a remote supervisor, the experimenters observe biological outcomes, but have no feedback on labels before the remote experimenter is aware of the rate of success. As a consequence, Prob (*direct*) = Prob (*reverse*); since Prob (*direct*) + Prob (*reverse*) = 1, then Prob (*direct*) = 1/2. In summary:

- Prob (*direct*) = 1 with local assessment of correlations;
- Prob (*direct*) = 1/2 with remote assessment of correlations.

**Fig. 5:**
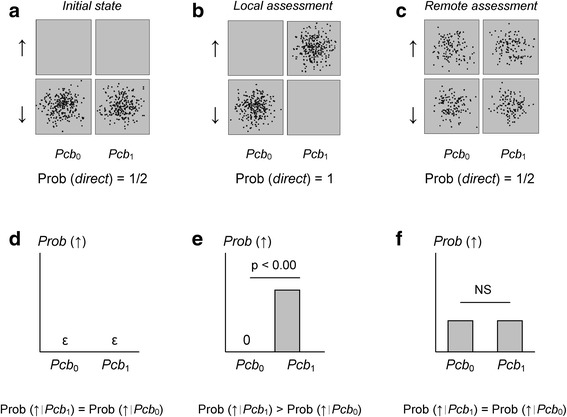
Comparison of local vs. remote assessment in an experiment with two placebos. In an experiment with a local assessment (local blind design or open-label experiment), correlations between labels (*Pcb*_0_ and *Pcb*_1_) and state﻿s of the﻿ bio﻿logical system (↓ and﻿ ↑) emerge (**b** and **e**) f﻿ro﻿m the initial state (**a** and **d**). These correlations vanish if the assessment of the experiment is made in a blind experiment with a remote supervisor (**c** and **f**). In this latter case, the difference between the biological changes associated with *Pcb*_0_ and *Pcb*_1_ is not statistically significant (NS) and the biological changes (“↑”) are randomly distributed among the two placebos. The difference of results in local vs. remote assessments offers the opportunity to test the modeling

Figure 5 illustrates the consequences of the assessment of the correlations with a remote assessment according to the modeling. In this case (blind experiment with an external supervisor), there is no statistical difference between the biological outcomes associated with *Pcb*_0_ and *Pcb*_1_ in contrast with a local assessment (local blind design or open-label experiment).

The experimental context is therefore crucial for establishing a relationship in the modeling. With a local assessment, the experimenters observe labels and then biological outcomes (open-label experiment) or observe biological outcomes and then labels (local blind experiments). In contrast, with a remote supervisor, the experimenters observe biological outcomes, but have *no feedback* on labels. If a local observer/experimenter is the *first* to assess the relationship, correlations emerge; if a remote supervisor is the *first* to assess the relationship, correlations vanish (biological changes are nevertheless observed, but at random places). Of course, in all cases, when participants met together, they agree on the conclusion (correlation or no correlation). The *order of the assessments* (local first or remote first) is the key element for the degree of correlation.

It is important to underscore that this difference between local and remote assessment of correlations offers the opportunity to test the modeling.

### Characterization of the role of the observers O and O’

The experimenters/observers *O* and *O’* play a crucial role in the modeling and we examine in this section how their involvement could be characterized and quantified.

As previously reported in Eq. (2), the joint probability of two independent events *A* and *B* is equal to the product of the separate probabilities of *A* and *B*. This equation can be generalized for two events *A* and *B* according to their degree of independence:

9 $$ \mathrm{Prob}\ \left( A\cap B\right) = \mathrm{Prob}\ (A) \times \mathrm{Prob}\ (B) + d\left(\mathrm{with}\ 0\ \le d\le\ 1\right) $$

**Fig. 6:**
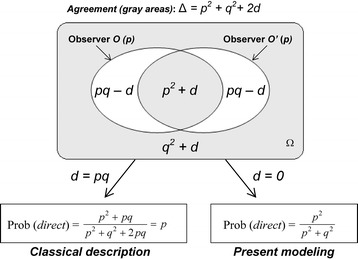
From a classical description of the experimental situation to the present modeling. The experimental situation depicted in Fig. 3 is generalized in this figure by using the parameter *d* which varies with the degree of independence of the probabilistic expectations on the outcome assigned to *O* and *O’*. The values of the two areas with impossible situations (direct relationship for one observer and reverse relationship for the other one) are calculated as: *p* – (*p* ^2^ + *d*) = *p ×* (1 – *p*) – *d* = *pq* – *d.* For *d* = 0, correlations between labels and biological outcomes emerge and, for *d = pq*, the probability of a direct relationship is equal to *p* as in classical probability. Ω, probability space

The degree of independence increases when the value of *d* decreases; the two events are completely independent with *d* = 0. In other words, the correlation of the two events increases when the value of *d* increases. Eq. (3) can be easily modified if *d* is taken into account (Fig. 6; see legend for calculation details):

10 $$ \mathrm{Prob}\ (direct)=\frac{p^2+ d}{p^2+{q}^2+2 d}\ \left(\mathrm{with}\ 0\le d\le pq\right) $$

When the parameter *d* varies from *d* = *pq* to *d* = 0, the experimental situation progressively shifts from a classical description to the present modeling (Fig. 6).

“Observing” an experiment requires a frame (what are we expecting?) and a feedback (what did we record?). Equation (6) indicates that there is no transition of Prob (*direct*) towards a stable position *in the absence of observers* (*N* = 0). We can draw the same conclusion if the observers are physically present in the laboratory, but not focusing their attention on this specific experiment (they expect nothing about the experimental system and they do not receive feedback). As a consequence, the parameter *d* can be considered as an evaluation of the attention of the team of experimenters to observe the predefined relationship between labels and biological outcomes. When *d* = 0, the observers are fully committed and for *d* = *pq* their attention is completely drawn away from the experiment. For intermediate values, the team is more or less occupied with these observations. Therefore, experimenters’ qualities, such as attention, commitment and persistence, appear to be necessary during the experiments for the emergence of correlations between labels and biological outcomes.

We can go a step further by considering that the parameter *d* is also an assessment of the capability of the experimenters to recognize (or not) the direct and reverse relationships per se, i.e. as new “objects” regardless their components, namely the association of the biological outcomes with *Pcb*_0_ and *Pcb*_1_. Indeed, in Eq. (4) (i.e. with *d* = 0), it is implicit that the experimenters recognize the outcome per se (i.e. in its “wholeness” or as such) as it would be the case for the outcome of a dice roll or the position of a pointer on a measurement device. But suppose now a team of experimenters *O* and *O’* who are inexperienced and do not recognize the predefined experimental relationship as a structured ensemble. The experimenters identify the sub-events as separate elements without integrating them as a whole (these sub-events are the association of the biological outcomes with *Pcb*_0_ and *Pcb*_1_). Since we continue to adopt the standpoint of *P*, we use Eq. (4) to calculate the evolution of the probability of each sub-event. Before the first fluctuation probability, the probabilities of the two sub-events are: Prob (*direct* | *Pcb*_0_) = 1 and Prob (*direct* | *Pcb*_1_) = 0 (see Fig. 5a). We notice that Prob (*direct* | *Pcb*_0_) and Prob (*direct* | *Pcb*_1_) are already in stable positions. Therefore, by using Eq. (4) (see also Fig. 4), these conditional probabilities are maintained in their respective stable positions with Prob (*direct* | *Pcb*_0_) that tends toward 1 and Prob (*direct* | *Pcb*_1_) that tends toward 0. The experimental results associated with these uncoupled sub-events can be gathered in order to calculate Prob (*direct*) by using the law of total probability:

11 $$ \mathrm{Prob}(direct)=\mathrm{Prob}\left( Pc{b}_0\right)\times \mathrm{Prob}\left( direct\Big| Pc{b}_0\right)+\mathrm{Prob}\left( Pc{b}_1\right)\times \mathrm{Prob}\left( direct\Big| Pc{b}_1\right) $$

12 $$ =1/2\times 1+1/2\times 0=1/2 $$

As a consequence, because the relationship between labels and biological outcomes is not recognized as a structured ensemble, there is no transition and Prob (*direct*) tends toward 1/2.

These considerations are reminiscent from Gestalt psychology that states that human mind spontaneously tends to perceive phenomena as structured ensembles (Gestalt) and not as a simple addition of parts. For example, the well-known Necker cube is immediately recognized as a 3D cube by a human observer and not as the simple addition of lines drawn on a 2D sheet [15]. We instantly “see” a cube in space because we have learned to perceive these 2D drawings as 3D “objects”.

As for Necker cube, cognitive and learning processes are undoubtedly at work for the passage from an “analytic” (*d* = *pq*) to a “structured” (or global) perspective (*d* = 0). In the first situation (*d* = *pq*), the experimenters are spectators of the experimental landscape that is perceived as a “collection of points”; in the second situation (*d* = 0), they are actors who interpret the experimental landscape that is perceived as a “form” [15]. In this last case, the experimenters concentrate their attention towards a “transcendent object” (namely, the predefined relationship) without reference to the details that become indiscernible.

If cognitive and learning processes are involved in the emergence of quantum-like correlations, different teams of experimenters with different training and experience should report various degrees of correlations between labels and biological outcomes in experiments comparing two placebos.

### Emergence of a quantum-like logic

Only tools from classic probability are used in the modeling. Nevertheless, as demonstrated in this section, there is an underlying quantum-like logic which is rooted in the initial partition of placebos as *Pcb*_*0*_ and *Pcb*_*1*_. Indeed, according to Fig. 1:

13 $$ \mathrm{Prob}\left( Pc{b}_0\right)\times \mathrm{Prob}\left(\downarrow \right)+\mathrm{Prob}\left( Pc{b}_0\right)\times \mathrm{Prob}\left(\uparrow \right)+\mathrm{Prob}\left( Pc{b}_1\right)\times \mathrm{Prob}\left(\downarrow \right)+\mathrm{Prob}\left( Pc{b}_1\right)\times \mathrm{Prob}\left(\uparrow \right)=1. $$

When the stable position #1 is achieved, Prob (*Pcb*_0_) = Prob (↓) and Prob (*Pcb*_1_) = Prob (↑) (see Fig. 5b); when the stable position #2 is achieved, Prob (*Pcb*_0_) = Prob (↑) and Prob (*Pcb*_1_) = Prob (↓). Therefore in both cases:

14 $$ {\left[\mathrm{Prob}\ \left( Pc{b}_0\right)\right]}^2+{\left[\mathrm{Prob}\ \left( Pc{b}_1\right)\right]}^2+2\times \mathrm{Prob}\ \left( Pc{b}_0\right)\times \mathrm{Prob}\ \left( Pc{b}_1\right)=1 $$

This equation is equivalent to:

15 $$ {\left[\mathrm{Prob}\ \left( Pc{b}_0\right)+\mathrm{Prob}\ \left( Pc{b}_1\right)\right]}^2=1 $$

Then, we define *a* and *b* such as Prob (*Pcb*_0_) = *a* ^2^ (or *a.a*) and Prob (*Pcb*_1_) = *b* ^2^ (or *b.b*). These definitions correspond to the stable position #1 (for the stable position #2, *b* ^2^ must be taken equal to –*b × −b*):

16 $$ {\left( a\cdot a+ b\cdot b\right)}^2={\left( a\cdot a\right)}^2+{\left( b\cdot b\right)}^2+2\times {\left( a\cdot b\right)}^2 = 1 $$

17 $$ {\left( a\cdot a+ b\cdot b\right)}^2+{\left( b\cdot a- a\cdot b\right)}^2={\left( a\cdot a\right)}^2+{\left( b\cdot b\right)}^2+{\left( b\cdot a\right)}^2+{\left( a\cdot b\right)}^2=1 $$

18 $$ 1 + 0 = 1/2 + 1/2 = 1 $$

**Fig. 7:**
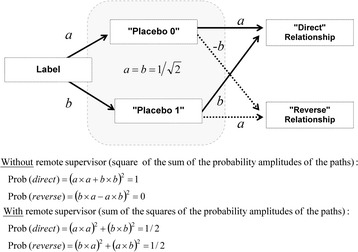
Probability of a direct relationship without or with a remote supervisor. The quantum-like probability of a direct relationship is calculated as the *square of the sum* of the probability amplitudes of the different possible “paths”. With a remote supervisor, classical probability applies and the probability of a direct relationship is calculated as the *sum of squares* of the probability amplitudes of the “paths”. Therefore, the probabilities of a direct relationship are different without or with a remote supervisor

As can be seen in Fig. 7, the left-hand side of Eq. (17) is the sum of Prob (*direct*) plus Prob (*reverse*) without a remote supervisor and the right-hand side is the sum of Prob (*direct*) plus Prob (*reverse*) with a remote supervisor. The terms *a* and *b* are thus *probability amplitudes* and their squaring allows calculating the corresponding probabilities.

Therefore, the probability of a direct relationship without a remote supervisor is calculated by doing the sum of the probability amplitudes of the two paths that lead to a direct relationship and then by squaring this sum. With a remote supervisor, the probability of a direct relationship is calculated by squaring the probability amplitude of each path that leads to a direct relationship and then by making the sum of the probabilities of the two paths (Fig. 7).

The relationship between labels and biological outcomes in the modeling has the same logic as single-photon self-interferences in Young’s double-slit experiment where photons behave either as particles when paths are detected or as waves when paths are not detected. In Fig. 7 that sketches an elementary experiment, quantum-like correlations are observed when “paths” (i.e. labels) are undistinguishable (from an outside standpoint) and correlations vanish when they are distinguishable for a remote supervisor. In this last case, each label is forced to adopt a defined “pathway”.

The emergence of quantum-like correlations is the consequence of the initial assumptions, namely the independent probabilistic expectations and the intersubjective agreement. The concomitant consideration of these two assumptions implies that the outcome of an experiment does not pre-exist to the interaction of *O* and *O’* from the standpoint of *P*. This is a characteristic of quantum measurements and, in the language of quantum mechanics, the “state” of *O* concerning his identification of the outcome would be said “superposed” before interacting with *O’* (and vice versa). The intersubjective agreement plays a similar role as a conservative law in physics and *O* and *O’* would be said “entangled” after their interaction.

### Importance of an uninvolved standpoint

The uninvolved standpoint of the participant *P* is central in the construction of the modeling. Indeed, from the standpoint of *O*, if he observes a direct relationship or a reverse relationship, then he can hold for sure that *O’* will tell him that he observes the same event. As a consequence the probability that *O* and *O’* observe a direct relationship is *p* in this case as stated by classical probability and not *p* × *p* (before renormalization) from the standpoint of *P*. The standpoints of *P* and *O-O’* coincide in situations where these two equations are verified:

19 $$ p=\frac{p^2}{p^2+{q}^2}\mathrm{and}\  q=\frac{q^2}{p^2+{q}^2} $$

These two equations are equivalent to (2*p* – 1) (*p* – 1) = 0 and (2*q* – 1) (*q* – 1) = 0, respectively. Therefore, there are only three possible values for *p*: 1/2, 1 or 0. These values are the probabilities associated with initial position, stable positions #1 and #2, respectively. Only the outside standpoint of *P* who is not involved in the observation of the experiment allows describing the transition of Prob (*direct*) from 1/2 to 1 (or 0) as a consequence of the emergence of quantum-like “interferences” (i.e. the cross-terms with probability amplitudes equal to *b* and *-b* in Fig. 7).

The differences between the standpoints of *O-O’* and *P* are the consequence of the demonstration of Breuer about the impossibility of a complete self-measurement [16]. According to this demonstration, a measurement apparatus (or an observer) is unable to distinguish all the states of a system in which it is contained (whether this system is classical or quantum mechanical does not matter). Only a second external apparatus (*P*) that observes both the first apparatus (*O*) and the system (*S*) is able to account all correlations between *O* and *S* [17].

### Optimized placebos in clinical trials

Without any doubt, the success of many complementary or alternative medicines rests on placebo effect. Thus, most authors consider homeopathy as a perfect illustration of the enforcement of the placebo effect in medicine. Moreover, homeopathic medicines could be considered as “super placebos” (or optimized placebos) since even practitioners think that they prescribe “true” medicines despite the absence of active molecules. Indeed, the manufacturing process of a majority of homeopathic medicines eliminates the initial active molecules by serially diluting them well beyond the limit set by Avogadro*’*s number. In other words, there are zero active molecules in these highly diluted samples. Even if tiny traces of the initial molecules would be present (due to contamination or imperfect diluting process), it remains to demonstrate how they could nevertheless have an effect contradicting the law of mass action.

Since no classical pharmacological action can be assigned to high dilutions, it has been suggested that modifications of water structure during the dilution process could account for the alleged effects. Until now, no convincing evidence has been reported indicating that modifications of water structure *specific* of the initial molecules are able to induce *specific* biologic changes. Moreover, homeopathy medicines available in pharmacies are sugar granules that have been impregnated with high dilutions and then dried. Therefore, until there is evidence to the contrary, the most reasonable scientific attitude is to consider homeopathy medicines and high dilutions as plain placebos.

Interestingly, the gold standard for drug evaluation, namely blind randomized clinical trial, appears to be an obstacle in studies aimed to establish the efficacy of homeopathy medicines. Thus, the study of Shang et al. compared homeopathy trials and matched conventional-medicine trials [18, 19]. The authors concluded that homeopathy medicines were comparable to placebos. Indeed, in contrast with conventional medicines, double-blind design was associated with a strong decrease of the probability of success when compared with open-label design. Although this study has been heavily criticized by proponents of homeopathy, most of them nevertheless acknowledge that blind randomized clinical trials are not adequate for assessing homeopathy medicines [20, 21]. A randomized clinical trial by Brien et al. in patients with rheumatoid arthritis suggested that homeopathy consultations, but not homeopathy medicines, were associated with a clinical benefit thus reinforcing the idea of a placebo effect [22].

In 2013, I proposed a slight modification of trial design in order to increase the chance to observe a difference between outcomes in double-blind placebo-controlled randomized trials of homeopathy medicines. This suggestion was not an encouragement for the practice of homeopathy, but an attempt to understand the persisting success of this alternative medicine in the absence of a rational basis. Based on the hypothesis that quantum-like correlations were responsible for “successful” open-label homeopathy clinical trials, it was proposed to replace the centralized assessment of efficacy in blind trials (generally done by statisticians) with a local assessment (by physicians) [23]. Thieves et al. recently challenged this hypothesis and reported experiments in a plant model (wheat germination) that compared a homeopathy medicine and a placebo both in local and centralized blind designs [24]. The results were in favor of the initial hypothesis since a significant difference of plant growth was observed between homeopathy medicine and placebo with local assessment while there was no significant difference with centralized assessment. The interaction test for local vs. centralized blind designs was statistically significant (p = 0.003). If we consider all samples (including homeopathy medicine) as plain placebos that differ only by their labels, these results are in favor of the present hypothetical modeling. These results should be also an encouragement for physicians to implement the same local blind design in clinical trials comparing a placebo with homeopathy medicine (i.e. a second placebo) in order to test in vivo the hypothesis of quantum-like correlations as depicted in Fig. 5.

## Discussion

It is generally thought that the macroscopic world escapes to the consequences of quantum physics due to the decoherence process. As a consequence, biological systems are considered to behave only classically. Nevertheless, some phenomena such as photosynthetic light harvesting or avian magnetoreception have been recently suggested to be the consequence of quantum phenomena [25]. Asano et al. evidenced quantum-like probabilistic behavior in *Escherichia coli* lactose-glucose metabolism [26]. In experimental psychology, some processes of cognition appear to obey to nonclassical logic [27]. Thus, the purpose of the new field named "quantum cognition” is to describe cognitive processes such as reasoning, decision making, judgment, language, memory or perception with mathematical quantum tools [28–31]. Moreover, Aerts described some experimental situations in physics where macroscopic devices could exhibit a quantum-like behavior [32]. Interestingly, Aerts showed that quantum probabilities could be introduced as the consequence of a lack of knowledge about fluctuations during the interaction between a measuring device and the object to be measured [32]. Most authors that use quantum probability outside the field of physics do not consider that the systems they describe are really quantum. Tools of quantum probability are simply used to describe results that until then were considered paradoxical [27]. Indeed, quantum physics is not only a new mechanics but also a new probability theory. An extension of classical probability with some mathematical tools borrowed to quantum probability (e.g. superposition, entanglement, interferences) appears to be fruitful in these different domains. With the present hypothetical modeling, it is proposed that quantum-like correlations could be a component of the placebo effect.

A central question is the generalisability of the proposed modeling to other experimental situations. Indeed, one could argue that bets on a coin toss could be also described by the same modeling by replacing labels with bets and biological system with coin toss. The answer is in Eq. (6) that supposes first that the system *S* has an internal structure submitted to small random fluctuations (thermal fluctuations for example) and second that each *p*_n+1_ value is strongly dependent on *p*_n_ value. In other words, probabilities *p*_n+1_ are correlated with probabilities *p*_n_. This last characteristic is named temporal autocorrelation and is a feature of phenomena with slow random fluctuations such as systems submitted to Brownian motion or biological systems. Of course, another implicit condition is the absence of physical obstacles that would block the transition of Prob (*direct*). Therefore, for systems based on a phenomenon not submitted to internal fluctuations (radioactive decay) or “rigid” systems with sufficient mechanical inertia to be not influenced (coin flipping or dice rolling), ε is equal to zero and no transition is possible. For experimental systems submitted to internal fluctuations, but with successive states that are not autocorrelated due to strong restoring forces (“elastic” systems), a transition as described in Fig. 4 is not possible (only random fluctuations around 1/2 are observed). An example of such a system is a beam splitter that randomly transmits or reflects a photon and vibrates around a fixed point. Systems based on phenomena with large random fluctuations (electronic noise for example) are also unsuitable.

The possibility to be experimentally tested is the hallmark of a scientific theory. The proposed modeling predicts that quantum-like correlations vanish when they are assessed by a remote supervisor. Only local assessments allow quantum-like “interferences” with correlation of “expected” and observed outcomes. It is important to emphasize that this modeling does not describe a causal relationship between mental states (e.g. intention) and physical states. Indeed, only quantum-like correlations are allowed and there is no way to transmit messages, instructions or orders from a laboratory to another one by using a series of coded samples.

Walach has extensively studied the relationship between homeopathy and notions from quantum logic such as complementarity and entanglement by using a “generalized quantum theory” [33, 34]. Of interest, this author insisted that homeopathy medicines and their associated clinical outcomes could not be treated causally (as it the case in blind randomized clinical trials), otherwise mismatches between outcomes occurred [35]. The present modeling with two placebo, which are differently labeled, leads to the same conclusion. Moreover, it is not excluded that quantum-like correlations could emerge in clinical trials for conventional drugs and add to classical causal relationship.

Some authors reported clinical trials where placebos associated with different labels or therapeutical rituals could lead to different outcomes [36, 37]. Only psychological mechanisms were supposed to be the cause of the different outcomes. Nevertheless, it would be interesting to evaluate a possible involvement of quantum-like correlations in such experiments aimed at investigating the placebo effect.

The potential existence of quantum-like correlations in the context of the experimenter effect could be also an element interesting to explore in the current debate about low reproducibility in life sciences [38]. Indeed, differences among experimenters’ teams are expected for the establishment of quantum correlations according to the modeling. As a matter of fact, trials in biology, medicine or psychology could benefit from an extended theory of probability that permits interferences between probabilities (more exactly between probability amplitudes).

## Conclusion

The hypothetical modeling proposed in this article suggests that two placebos with different labels can be associated with different outcomes even in blind trials. Such a counterintuitive conclusion is the consequence of a probabilistic modeling that authorizes quantum-like interferences. This modeling could give a framework for some unexplained observations where mere placebos are compared (in some alternative medicines for example) and could be tested in blind trials by comparing local vs. remote assessment of correlations.
